# Miscellany

**Published:** 1903-02

**Authors:** 


### MISCELLANY.

---

The Tenth Annual Report of the Children's Hospital, of Buffalo, for the year ending October, 1902, has been issued. During this period 170 children have been cared for, of which number 93 have been cured and 49 relieved. An incubator ward has been established and two infant incubators have been installed after the pattern in use at the Pan-American Exposition, costing about $1,500. This splendid charity deserves well of our citizens.

---

Examinations of candidates for appointment in the medical corps of the U. S. Army will be resumed by the Army Medical Board in Washington, D. C., April 20, 1903. Classes will be invited to appear April 20, and each Monday thereafter as long as is necessary. Full information as to method of application, nature and scope of examination, and the like, will be furnished by the Surgeon-General at Washington upon request of those interested. Applicants from civil life are restricted in age to twenty-nine years, and hospital training or professional experience in private practice is expected of all candidates. There are at present thirty-five vacancies to be filled.

---

Civil Service Examination—Erie County Service.—An open competitive examination for the Erie County service will be held in Buffalo, February 7, 1903, for the positions mentioned below. Intending competitors must fill out application blanks and file them in the office of the commission on or before noon of February 5. Candidates having applications on file will be given ample notice of the time and place of examination. No one will be admitted to the examination without the official notice (form E-8) from the Chief Examiner of the Commission.

Competitors must be at least twenty-one years of age, and must be citizens and residents of Erie County. The time allowed for the examination is six hours.

Positions and requirements: physician, Erie County Penitentiary, $600; physician, Erie County Jail, $300. Candidates must be licensed medical practitioners of the State of New York. Subjects of examination and relative weights: general medicine and surgery, 6; questions on the organisation and administration of medical department, 2; experience, 2. For application blank, address Chief Examiner, State Civil Service Commission, Albany, N.Y.

